# Estimating the Characteristics and Emission Factor of Ammonia from Sewage Sludge Incinerator

**DOI:** 10.3390/ijerph18052539

**Published:** 2021-03-04

**Authors:** Seongmin Kang, Joonyoung Roh, Eui-Chan Jeon

**Affiliations:** 1 Climate Change & Environment Research Center, Sejong University, Seoul 05006, Korea; smkang9804@gmail.com; 2 Department of Climate and Environment, Sejong University, Seoul 05006, Korea; shdod88@naver.com

**Keywords:** PM 2.5 secondary sources, sewage sludge incinerator, uncertainty, missing sources, ammonia emission factor

## Abstract

In the case of sewage sludge, as direct landfilling was recently prohibited, it is treated through incineration. Among the air pollutants discharged through the incineration of sewage sludge, NOx and SOx are considered secondary substances of PM2.5 and are being managed accordingly. However, NH_3_, another of the secondary substances of PM2.5, is not well managed, and the amount of NH_3_ discharged from sewage sludge incineration facilities has not been calculated. Therefore, in this study, we sought to determine whether NH_3_ is discharged in the exhaust gas of a sewage sludge incineration facility, and, when discharged, the NH_3_ emission factor was calculated, and the necessity of the development of the emission factor was reviewed. As a result of the study, it was confirmed that the amount of NH_3_ discharged from the sewage sludge incineration facility was 0.04 to 4.47 ppm, and the emission factor was calculated as 0.002 kg NH_3_/ton. The NH_3_ emission factor was compared with the NH_3_ emission factor of municipal solid waste proposed by EMEP/EEA (European Monitoring and Evaluation Programme/European Environment Agency) because the NH_3_ emission factor of the sewage sludge incineration facility had not been previously determined. As a result of the comparison, the NH_3_ emission factor of EMEP/EEA was similar to that of municipal solid waste, confirming the necessity of developing the NH_3_ emission factor of the sewage sludge incineration facility. In addition, the evaluation of the uncertainty of the additionally calculated NH_3_ emission factor was conducted quantitatively and the uncertainty range was presented for reference. In the future, it is necessary to improve the reliability of the NH_3_ emission factor of sewage sludge incineration facilities by performing additional analysis with statistical representation. In addition, the development of NH_3_ emission factors for industrial waste incineration facilities should be undertaken.

## 1. Introduction

In South Korea, landfill and land reclamation of sewage sludge were prohibited in 2003. Furthermore, following the 1996 revision of the London Convention, sea dumping of sewage sludge was entirely prohibited in 2011 [1]. Incineration of sewage sludge offers the advantages of stable and sanitary operation along with the potential of utilizing incineration waste heat as an energy source. Hence, incineration is currently employed to treat sewage sludge in various countries, including Germany and Japan [2,3,4]. In sewage sludge incineration facilities, selective non-catalytic reduction (SNCR) is utilized to reduce the emission of nitrogen oxides (NOx). In the case of SNCR, NOx can be reduced, but if excessively operated, NH_3_ will be generated and discharged into the atmosphere [5,6,7,8]. NH_3_ is one of the secondary products of ultrafine particles and is considered to be an odorous air pollutant [9,10,11]. In the case of NH_3_, it is managed as an odor and air pollutant, but the concentration of emission-permissible regulations is high, and related emission management and research are insufficient. Considering the paucity of research on the management and emission control of ammonia, studies are required to estimate ammonia emissions and develop an emission factor.

In South Korea, both emission factors and emission concentrations of NH_3_ from sewage sludge incineration facilities have not yet been studied. Moreover, several other countries do not monitor or record NH_3_ emitted from sewage sludge incineration facilities in their air pollutant inventories [12]. This study aimed to analyze ammonia emissions through actual measurements and identify the necessity of determining the emission factor of ammonia for sewage sludge incineration facilities by determining the emission characteristics and emission factors along with their associated uncertainty.

## 2. Materials and Methods

### 2.1. Selection of Incineration Facilities

In this study, ammonia samples were collected from the flue gas emitted during sewage sludge incineration in order to check whether ammonia was discharged from the sewage sludge incineration facilities, and the concentration of NH_3_ in the collected samples was analyzed. Moreover, the target facilities were sewage sludge incineration facilities in South Korea. The operating conditions of the target facility and the number of samples collected are shown in Table 1. A total of 40 and 25 samples were collected from incineration in sewage sludge incinerator A and sewage sludge incinerator B, respectively.

### 2.2. Analysis of Ammonia

**Figure 1:**
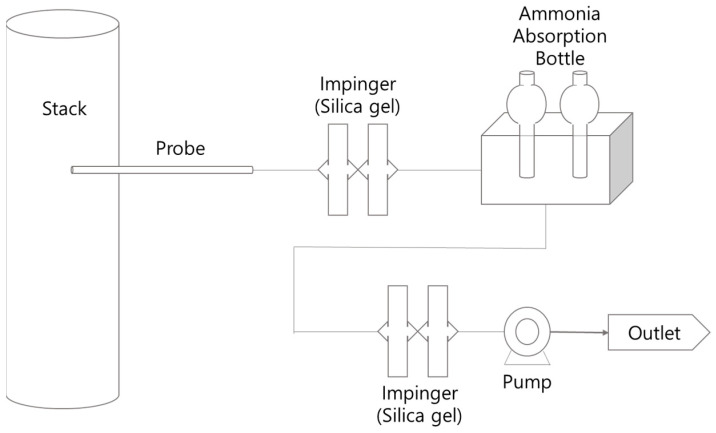
Schematic of the field setup for ammonia sampling in a sewage sludge incinerator.

In this study, the indophenol method, suggested by the “air pollution process test method” and “odor process test method” (which are used as the standard test methods in South Korea), was utilized for measuring the concentration of NH_3_ in the samples collected from the flue gas outlet of the sewage sludge incineration facilities [13,14]. The indophenol method measures the absorbance of indophenols generated through the reaction with ammonium ions after adding sodium hypochlorite and sodium phenol-nitroprusside solutions into the ammonia absorbent collected from the exhaust gas outlet. The sample collection procedure for ammonia analysis is as follows. First, a boric acid solution was placed in two 50-mL flasks for ammonia absorption. Subsequently, using a mini-pump (SIBATA MP-ΣNII, Saitama, Japan) operated for 20 min at a pumping rate of 4 L/min, 80 L of exhaust gas was absorbed into the boric acid. To remove the moisture present in the exhaust gas emitted during sewage sludge incineration, the absorption flasks used for sample collection were installed at the front end of the sample collection apparatus and silica gel was placed inside the flask [15]. Figure 1 shows a schematic diagram of the ammonia sample collection apparatus used in this study. Ammonia concentration was determined using a spectrophotometer (Shimadzu 17A, Kyoto, Japan) by measuring absorbance at a wavelength of 640 nm.

### 2.3. Development of NH_3_ Emission Factor

The unit of emission factor for gaseous emission substances at incineration facilities is used to express the amount of emission compared to the amount of material incineration. Therefore, after calculating the amount of discharge by considering the generally discharged gas concentration and the flow rate of the discharged gas, the emission factor is determined by considering the combustion amount of the exhaust gas generating source [16,17]. Therefore, in this study, the calculation of the ammonia emission factor from the sewage sludge incineration facility was based on the formula used in the related previous study, and the calculation formula takes the form of Equation (1) [18,19]. To calculate the NH_3_ emission factor, data on flow, concentration, and sewage sludge incineration volume are required. One-day cumulative CleanSYS data recorded at the target workplace in real-time were utilized as flow data. CleanSYS measures the flow and temperature of exhaust gases, including sulfur oxides, NOx, and particulate matter, at 5-min intervals using an air pollution monitoring system managed in South Korea [20]. Based on the incineration volume, data from the target workplace were utilized.

(1) $$EF_{{\mathrm{NH}}_{3}}=\left[C_{{\mathrm{NH}}_{3}}\times \frac{M_{w}}{V_{m}}\times Q_{day}\times 10^{-6}\right]/FC_{day}$$

where EF is the emission factor (kg NH_3_/ton); $C_{{\mathrm{NH}}_{3}}$ is the NH_3_ concentration in flue gas (ppm); $M_{w}$ is the molecular weight of NH_3_ (constant) = 17.031 (g/mol); $V_{m}$ is one mole ideal gas volume in standardized conditions (constant) = 22.4 (10^−3^ m^3^/mol); $Q_{day}$ is the daily accumulated flow rate (Sm^3^/day) (based on dry combustion gas); and $FC_{day}$ is the daily sewage sludge incineration (ton/day).

### 2.4. Uncertainty Analysis Using Monte Carlo Simulation

**Figure 2:**
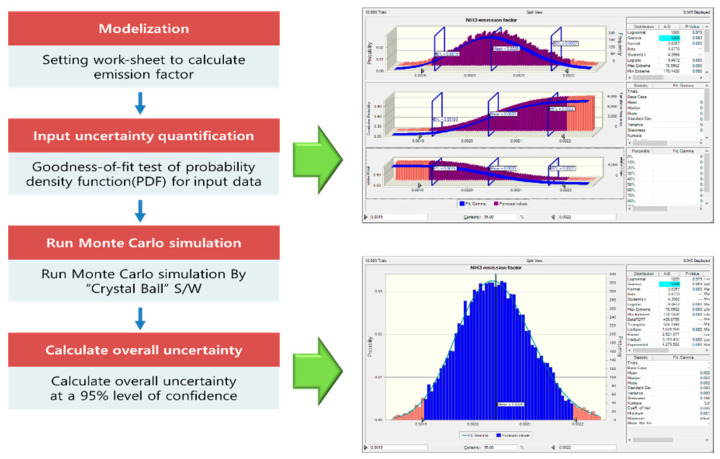
Process of the Monte Carlo simulation for estimating the uncertainty of the NH_3_ emission factor.

In this study, Monte Carlo simulation was used to calculate the uncertainty of the NH_3_ emission factor of the sewage sludge incineration facility. Monte Carlo simulation is a four-step procedure, as shown in Figure 2. In the first step, the model to be applied to the emission factor and the worksheet to be used in the estimation of emission factors were selected. In the second step, the probability density function of the input variable applied in the development of emission factor was verified through a suitability verification method. The significance level for the hypothesis testing was set at 95%. The data on NH_3_ emission concentration, incineration volume, and emission flow were subjected to suitability verification and the probability density function for each was selected. In the third step, Monte Carlo simulation and random sampling simulation were performed using Crystal ball; ver. 11.1.2.4 (Oracle Crystal ball, Oracle, Redwood City, CA, USA). Crystal Ball provides a system to analyze the Monte Carlo simulation using probability distributions based on a Microsoft Excel spreadsheet and graphically presents the types of probability distribution functions resulting from random sampling results [21].The fourth step was to confirm the distribution through the simulated results and calculate the uncertainty range, which was carried out with a 95% confidence interval.

## 3. Results and Discussion

### 3.1. NH_3_ Emission Concentration from Sewage Sludge Incinerators

Ammonia concentrations of the samples collected from the outlets of the two incineration facilities are listed in Table 2. The ammonia concentration from sewage sludge incinerator A ranged from 0.04 to 4.47 ppm, with a mean concentration of 1.28 ppm and standard deviation of 1.12 ppm. The ammonia concentration from sewage sludge incinerator B ranged from 0.07 to 3.22 ppm, with a mean concentration of 0.39 ppm and standard deviation of 0.72 ppm.

### 3.2. NH_3_ Emission Factor from Sewage Sludge Incinerators

The ammonia emission factors of the sewage sludge incineration facilities are listed in Table 3. The NH_3_ emission factor of the sewage sludge incineration facility was 0.002 kg NH_3_/ton. Currently, in South Korea, the emission factor of ammonia is not applied to sewage sludge incineration. Moreover, due to a lack of studies conducted in other countries, the results of this study cannot be compared with any existing studies of a similar nature. Therefore, the results were compared with the ammonia emission factor of a municipal solid waste incineration facility to identify the level of the estimated emission factor. The emission factor of the sewage sludge incineration facility was lower than that of the municipal solid waste, 0.009 kg NH_3_/ton, which has been calculated in South Korea. The emission calculated in this study was also lower than the EMEP/EEA(2016) [22]. Although these differences can be attributed to the difference in the type of waste, they can be also attributed to the difference in the type of incineration. In fluidized bed incineration, lower temperatures are used compared to stoker-type incineration, resulting in lower NOx emissions [23,24]. Therefore, the amount of ammonia used to reduce NOx as well as the ammonia slip generated is expected to be low. This explains the lower ammonia emission factor compared with the stoker-type incineration method. Although the emission factor calculated in this study is low, owing to ammonia emissions from these facilities, it needs to be included in emission inventories as an omitted emission source.

### 3.3. Uncertainty Analysis of NH_3_ Emission Factor from Sewage Sludge Incinerators

**Figure 3:**
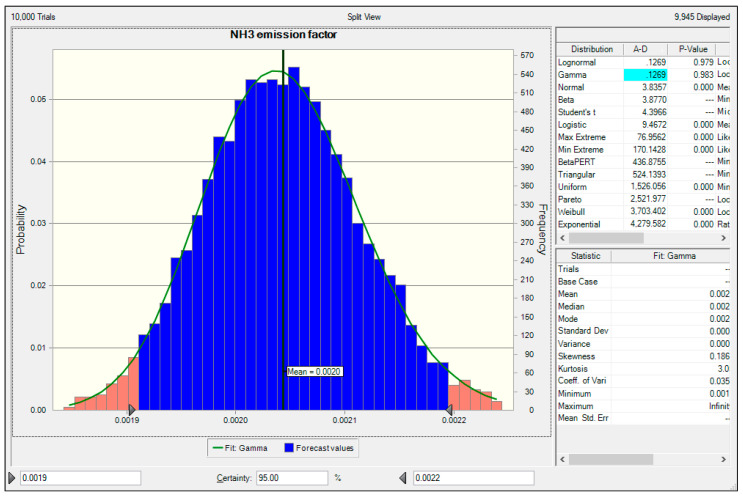
Process of the Monte Carlo simulation for estimating the uncertainty of the NH_3_ emission factor.

The results of the Monte Carlo simulation are presented in Figure 3. The probability density function of the NH_3_ emission coefficient of the sewage sludge incineration facility developed in this study was analyzed as Gamma distribution. The mean value was 0.020 kg NH_3_/ton, and at a 95% confidence level, the lower 2.5% and upper 97.5% were 0.0019 kg NH_3_/ton and 0.0022 kg NH_3_/ton, respectively. The uncertainty of the NH_3_ emission factor calculated using these values ranged between –5% and +10% at a 95% confidence level. Because the value and range of NH_3_ uncertainty have not been previously reported, the results of this study cannot be compared with those of others. However, the uncertainty of the NH_3_ emission factor for a thermal power plant as a stationery combustion facility was reported by Kang et al. (2020). According to Kang et al., the uncertainty of the NH_3_ emission factor estimated at a bituminous coal-fired power plant varied from −6.9% to +10.34% [5]. Hence, the uncertainty range of the emission factor calculated in this study was found to be lower.

In South Korea, the uncertainty evaluation of air pollutants is carried out using the rank method proposed by the U.S. EPA(United States Environmental Protection Agency) and evaluated through expert judgment [25,26]. In the case of the greenhouse gas emission factor, the uncertainty is mentioned and presented as a quantitative number [27]. Therefore, it is judged that air pollutants can be evaluated quantitatively in the future if they present an uncertainty range as with greenhouse gases.

## 4. Conclusions

In this study, the NH_3_ emission factor was calculated using the actual NH_3_ emission concentrations of sewage sludge incineration facilities. Furthermore, it was verified that the NH_3_ emission factor should be included in emission inventories as an omitted emission source. As a result of the study, NH_3_ was also emitted from the sewage sludge incineration facility, and the NH_3_ emission factor of the sewage sludge was calculated as 0.002 kg NH_3_/ton. It was confirmed that this emission factor was similar to the NH_3_ emission factor for domestic waste proposed by EMEP/EEA (2016). In addition, this study analyzed the uncertainty of the NH_3_ emission factor using the Monte Carlo model. As a result of the uncertainty analysis, it was confirmed that the probability density function of the NH_3_ emission factor of the sewage sludge incineration facility is Gamma distribution, and the uncertainty range is −5% to +10% at the 95% confidence level.

The contents and meanings that can be confirmed through this study are as follows.

1. The concentration of NH_3_ discharged from a sewage sludge incineration facility that is not currently calculating NH_3_ emissions was confirmed, and the NH_3_ emission factor was calculated and presented.
2. Currently, for sewage sludge incineration facilities, the U.S. EPA and European EMEP/EEA air pollution inventory do not present the NH_3_ emission factor of sewage sludge incineration facilities, but in the case of EMEP/EEA, only the NH_3_ emission factor of the MSW(Municipal Solid Waste) incinerator is presented. In this study, the NH_3_ emission factor of the sewage sludge incineration facility was calculated and compared with the emission factor of the MSW incineration facility of EMEP/EEA. As a result of the comparison, it was confirmed that the level was similar, and we then suggested the necessity of developing the NH_3_ emission factor for sewage sludge incineration facilities.
3. In the U.S. EPA and in Korea, the uncertainty of the emission factor is evaluated by expert judgment. However, EMEP/EEA in Europe introduces the uncertainty evaluation method currently used in the greenhouse gas inventory regarding uncertainty and presents the distribution of emission factors at the 95% confidence interval. In the case of EMEP/EEA, the uncertainty range is presented similarly, but the detailed level related to the uncertainty is not presented. In this study, uncertainty was evaluated by using Monte Carlo simulation, one of the uncertainty evaluation methods suggested by EMEP/EEA, for the calculated NH_3_ emission factor of the sewage sludge incineration facility, and we also presented the uncertainty. Therefore, it is presented so that related researchers can confirm the quantitative uncertainty.

In this study, the emission factor was calculated by targeting only two facilities to identify NH_3_ emission; hence, to increase the reliability of the emission factor, multiple facilities need to be included in future studies. Furthermore, the development of emission factors is expected to be required for facilities incinerating types of industrial waste other than municipal solid waste in the future. Therefore, a more reliable foundation can be formed if emission characteristics and emission factor uncertainty are analyzed.

## Figures and Tables

**Table 1:** Characteristics of the investigated sewage sludge incinerator in South Korea.

Site	Capacity	Type	Sampling
Sewage Sludge Incinerator A	90 ton/day	Fluidized bed	40
Sewage Sludge Incinerator B	50 ton/day	Fluidized bed	25

**Table 2:** NH_3_ concentration of the investigated sewage sludge incinerators.

Site	Mean (ppm)	Min (ppm)	Max (ppm)	SD (Standard Deviation)	Sampling
Sewage Sludge Incinerator A	1.28	0.04	4.47	1.12	40
Sewage Sludge Incinerator B	0.39	0.07	3.22	0.72	25

**Table 3:** Comparison of NH_3_ emission factor at the waste incinerator.

Classification	Waste Type	Incinerator Type	NH_3_ Emission Factor (kg NH_3_/ton)
This study	Sewage Sludge	Fluidized bed	0.002
Kang et al. (2020)	MSW (Municipal Solid Waste)	Stoker	0.009
EMEP/ EEA (2016) [22]		-	0.003

## Data Availability

Date sharing not applicable.

## References

[1] International Maritime Organization (1996). Convention on the Prevention of Marine Pollution by Dumping of Wastes and Other Matter.

[2] Fytili D., Zabaniotoua A. (2008). Utilization of sewage sludge in EU application of old and new methods—A review. Renew. Sustain. Energy Rev..

[3] Werther J., Ogada T. (1999). Sewage sludge combustion. Prog. Energy Combust. Sci..

[4] Takahiro M., Yoshizo S., Hidekazu N., Takafumi Y., Takami K., Hitoshi H., Seiichiro O. (2009). Combustion characteristics of sewage sludge in an incineration plant for energy recovery. Fuel Process. Technol..

[5] Seongmin K., Seong-Dong K., Eui-Chan J. (2020). Emission Characteristics of Ammonia at Bituminous Coal Power Plant. Energies.

[6] Liu B., Yan F., Hu J., Turkson R., Lin F. (2016). Modeling and Multi-Objective Optimization of NOx Conversion Efficiency and NH_3_ Slip for a Diesel Engine. Sustainability.

[7] Wielgosinski G., Czerwinska J., Szymanska O., Bujak J. (2020). Simultaneous NOx and Dioxin Removal in the SNCR Process. Sustainability.

[8] Seongmin K., Ji-yun W., Eui-Chan J. (2020). Ammonia Emission Characteristics and Emission Factor of Municipal Solid Waste Incineration Plant. Sustainability.

[9] Ministry of Environment (2016). Fine Dust, What Is It?.

[10] Ministry of Environment (2017). Management Strategies to Reduce PM-2.5 Emission: Emphasis-Ammonia.

[11] Environmental Preservation Association (2015). POLICY & ISSUES Environment column: Air Pollutant Total Management System. Environ. Inf..

[12] NIER (National Institute of Environmental Research in Korea) (2008). National Air Pollutant Emission Estimation Manual (III).

[13] Ministry of Environment in Korea (2019). Standard Methods for the Measurements of Air Pollution.

[14] Ministry of Environment in Korea (2019). Standard Method of Odor Compounds.

[15] Wujie W., Yu X., Haochang S., Xiaojuan H., Keng Y., Guoliang W., Yucheng C. (2020). Characteristics of Ammonia Removal and Nitrifying Microbial Communities in a Hybrid Biofloc-RAS for Intensive Litopenaeus vannamei Culture: A Pilot-Scale Study. Water.

[16] United States Environmental Protection Agency (EPA) (1997). Recommended Procedures for Development of Emissions Factors and Use of the WebFIRE Database.

[17] United States Environmental Protection Agency (EPA) (2013). AP-42: Compilation of Air Emissions Factors.

[18] Seongmin K., Yoon-jung H., Seong-Dong K., Eui-Chan J. (2020). Ammonia Emission Factors and Uncertainties of Coke Oven Gases in Iron and Steel Industries. Sustainability.

[19] Seongmin K., Yoon-jung H., Eui-Chan J. (2020). Ammonia Emission Sources Characteristics and Emission Factor Uncertainty at Liquefied Natural Gas Power Plants. Int. J. Environ. Res. Public Health.

[20] Daejeon Sejong Research Institute (2019). Management Plan for Particulate Matter Reduction at Autonomous Agreement Air Pollutants-Reducing Sites.

[21] Oracle (2014). Oracle Crystal Ball Spreadsheet Functions for Use in Microsoft Excel Models.

[22] European Environment Agency (2016). EMEP/CORINAIR Atmospheric Emission Inventory Guidebook.

[23] De Winter P., Cahusac P.M. (2014). Starting Out in Statistics: An Introduction for Students of Human Health, Disease, and Psychology.

[24] Gibbons J.D., Chakraborti S. (2003). Nonparametric Statistical Inference Fourth Edition, Revised and Expanded.

[25] Emission Inventory Improvement Program (EIIP) (1996). Recommended Approach to Using the Data Attribute Rating System (DARS), EIIP Technical Report Series Volume 6, Appendix F.

[26] Emission Inventory Improvement Program (EIIP) (1996). Technical Report Series Volume 6, Evaluating the Uncertainty of Emission Estimates.

[27] IPCC (2006). The 2006 IPCC Guidelines for National Greenhouse Gas Inventories. General Guidance and Reporting.

